# Epigenetic Inactivation of the Tumor Suppressor *IRX1* Occurs Frequently in Lung Adenocarcinoma and Its Silencing Is Associated with Impaired Prognosis

**DOI:** 10.3390/cancers12123528

**Published:** 2020-11-26

**Authors:** Miriam M. Küster, Marc A. Schneider, Antje M. Richter, Sarah Richtmann, Hauke Winter, Mark Kriegsmann, Soni S. Pullamsetti, Thorsten Stiewe, Rajkumar Savai, Thomas Muley, Reinhard H. Dammann

**Affiliations:** 1 Faculty of Biology, Institute for Genetics, Justus-Liebig-University Giessen, 35392 Giessen, Germany; Miriam.Kuester@gen.bio.uni-giessen.de (M.M.K.); antje.m.richter@gen.bio.uni-giessen.de (A.M.R.); 2 Translational Research Unit, Thoraxklinik at Heidelberg University Hospital, 69126 Heidelberg, Germany; Marc.Schneider@med.uni-heidelberg.de (M.A.S.); Sarah.Richtmann@med.uni-heidelberg.de (S.R.); Thomas.Muley@med.uni-heidelberg.de (T.M.); 3 Marburg Lung Center (UGMLC) and Translational Lung Research Center (TLRC) Heidelberg, German Center for Lung Research (DZL), Universities of Giessen, 35392 Giessen, Germany; Hauke.Winter@med.uni-heidelberg.de (H.W.); mark.kriegsmann@med.uni-heidelberg.de (M.K.); soni.pullamsetti@mpi-bn.mpg.de (S.S.P.); thorsten.stiewe@staff.uni-marburg.de (T.S.); Rajkumar.Savai@mpi-bn.mpg.de (R.S.); 4 Department of Surgery, Thoraxklinik at Heidelberg University Hospital, 69126 Heidelberg, Germany; 5 Department of Pathology, Institute of Pathology, University Hospital Heidelberg, 69120 Heidelberg, Germany; 6 Department of Lung Development and Remodeling, Max-Planck-Institute for Heart and Lung Research, 61231 Bad Nauheim, Germany; 7 Institute of Molecular Oncology, Member of the German Center for Lung Research (DZL), Philipps-University, 35032 Marburg, Germany

**Keywords:** IRX, DNA methylation, epigenetic, lung cancer, adenocarcinoma, apoptosis, tumor suppressor, homeobox

## Abstract

**Simple Summary:**

Lung cancer is one of the most commonly diagnosed cancers worldwide and the most common cause of cancer-related deaths. During lung carcinogenesis, epigenetic alteration of tumor-related genes is a frequent event and especially silencing of tumor suppressor genes is often found. In our work, we identified *Iroquois homeobox 1* (IRX1) from the lung cancer susceptibility locus 5p15.33, as an epigenetically silenced target gene. We report frequent epigenetic inactivation of *IRX1* in primary lung adenocarcinoma. Moreover, reduced expression and hypermethylation of *IRX1* was correlated with an impaired prognosis of patients with lung adenocarcinoma. Functionally, IRX1 overexpression induced signs of apoptosis including fragmented nuclei and expression of a proapoptotic regulator. Loss of *IRX1* expression by its promoter hypermethylation can serve as a diagnostic and prognostic lung cancer biomarker.

**Abstract:**

*Iroquois homeobox* (IRX) encodes members of homeodomain containing genes which are involved in development and differentiation. Since it has been reported that the *IRX1* gene is localized in a lung cancer susceptibility locus, the epigenetic regulation and function of IRX1 was investigated in lung carcinogenesis. We observed frequent hypermethylation of the *IRX1* promoter in non-small cell lung cancer (NSCLC) compared to small cell lung cancer (SCLC). Aberrant *IRX1* methylation was significantly correlated with reduced *IRX1* expression. In normal lung samples, the *IRX1* promoter showed lower median DNA methylation levels (<10%) compared to primary adenocarcinoma (ADC, 22%) and squamous cell carcinoma (SQCC, 14%). A significant hypermethylation and downregulation of *IRX1* was detected in ADC and SQCC compared to matching normal lung samples (*p* < 0.0001). Low *IRX1* expression was significantly correlated with impaired prognosis of ADC patients (*p* = 0.001). Reduced survival probability was also associated with higher *IRX1* promoter methylation (*p* = 0.02). Inhibition of DNA methyltransferase (DNMT) activity reactivated *IRX1* expression in human lung cancer cell lines. Induced DNMT3A and EZH2 expression was correlated with downregulation of *IRX1*. On the cellular level, IRX1 exhibits nuclear localization and expression of IRX1 induced fragmented nuclei in cancer cells. Localization of IRX1 and induction of aberrant nuclei were dependent on the presence of the homeobox of IRX1. By data mining, we showed that *IRX1* is negatively correlated with oncogenic pathways and *IRX1* expression induces the proapoptotic regulator *BAX*. In conclusion, we report that *IRX1* expression is significantly associated with improved survival probability of ADC patients. *IRX1* hypermethylation may serve as molecular biomarker for ADC diagnosis and prognosis. Our data suggest that *IRX1* acts as an epigenetically regulated tumor suppressor in the pathogenesis of lung cancer.

## 1. Introduction

Lung cancer is one of the most commonly diagnosed cancers worldwide (1.82 million in 2012) and the most common cause of cancer-related deaths (1.6 million in 2012) [1]. Lung cancer is classified into small cell lung cancer (SCLC) and non-small cell lung cancer (NSCLC) which is further subdivided into different entities including adenocarcinoma (ADC), squamous cell carcinoma (SQCC), and large cell carcinoma (LC). Whereas SCLC originates from neuroendocrine cells of the lung, ADC and SQCC arises from epithelial cells that undergo distinct genetic and epigenetic changes. During lung carcinogenesis, epigenetic alteration of tumor-related genes is a frequent event and especially silencing of tumor suppressor genes is often found [2]. The hallmark of the epigenetic inactivation is the hypermethylation of CpG island promoters through 5-methyl-cytosine that leads to chromatin silencing of the corresponding gene [3]. In our previous work, we identified several tumor suppressor genes (e.g., *RASSF1A*, *RASSF10*, *ABCB4*, *DUSP2*, and *ZAR1*) that are epigenetically silenced by DNA hypermethylation in human carcinogenesis including lung tumors [4].

Homeobox containing genes have been shown to play a central role in embryonic development, in cellular differentiation, and in pathogenesis of cancer [5,6]. In drosophila, the *Iroquois* gene (IRO) encodes a prepattern gene that controls the formation of bristles on the thorax [7]. In human, the *Iroquois homeobox* (IRX) family consists of six members (IRX1-IRX6) that contain a homeobox and a so called IRO box [8]. The *IRX* gene are transcription factors that play key roles in differentiation and development [9,10]. These six *IRX* genes are organized into two clusters of three, each are located on cytoband 5p15.33 (*IRX1*, *IRX2*, and *IRX4*) and on cytoband 16q12.2 (*IRX3, IRX5*, and *IRX6*) [5,8].

**Figure 1:**
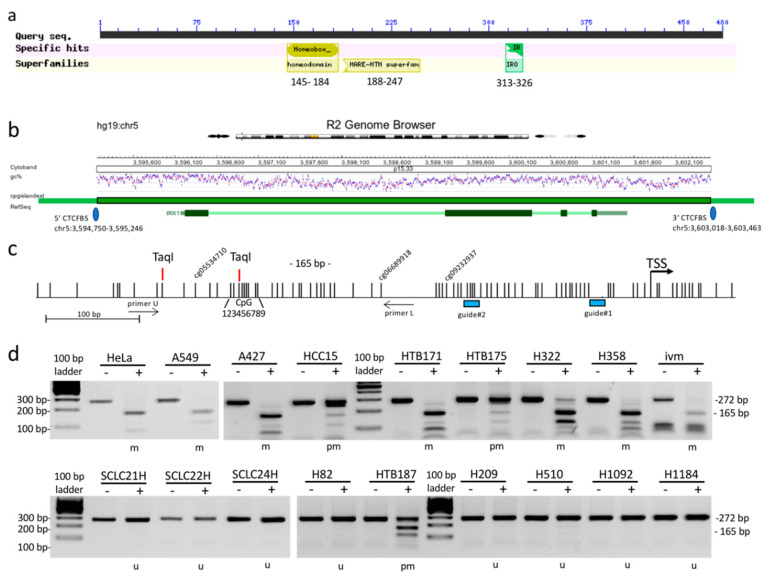
Structure of IRX1 and its methylation analysis in lung cancer. (**a**) The *IRX1* gene encodes a polypeptide of 480 aa and the protein contains a homeobox at position 145–184, a HARE-HTH (HB1, ASXL, restriction endonuclease helix-turn-helix) domain at position 188–247 and the Iroquois (IRO) box at position 313–326 (NCBI tool for conserved domain search) [26] (**b**) *IRX1* is located on chromosome 5p15.33 in an 8.6 kb CpG island (dark green box) flanked by the two indicated CTCF binding sites (5′- and 3′-CTCFBS) [27]. (**c**) Overview of the analyzed 675 bp sequence located in the proximal *IRX1* promoter region. The transcriptional start site (TSS; arrow) and individual CpG sites (black lines) are depicted by Python vs. CoBRA [28]. For methylation analysis, two *Taq*I sites of CoBRA, nine CpGs sites (1, 2, 3, 4, 5, 6, 7, 8, and 9) of bisulfite pyrosequencing, and three CpG sites (cg05534710, cg06689918, and cg09232937) of 450K array are marked. Positions of guide#1 and guide#2 for promoter deletion are marked as blue boxes upstream of the *IRX1* TSS. (**d**) A 272 bp fragment of the *IRX1* promoter was analyzed by CoBRA in five NSCLC (A549, A427, HCC15, H322, and H358), 11 SCLC (HTB171, HTB175, SCLC21H, SCLC22H, SCLC24H, H82, HTB187, H209, H510, H1092, and H1184), HeLa and in vitro methylated DNA (ivm). PCR products were mock (−) or *Taq*I (+) digested and analyzed on 2% agarose gels together with a 100 bp ladder. Unmethylated (u), partially methylated (pm), and methylated (m) samples are indicated.

The *IRX1* gene encodes a polypeptide of 480 amino acids (aa) and the protein contains a homeobox at position 145–184, a HB1, ASXL, restriction endonuclease helix-turn-helix (HARE-HTH) domain (position 188–247) and the IRO box at position 313–326 (Figure 1a). As mentioned above, *IRX1* is located on cytoband 5p15.33 and the loss of this chromosomal region (LOH; loss of heterozygosity) has been reported in different tumor types including gastric cancer [11], cervix carcinoma [12], colorectal cancer [13], and lung cancer [14]. The promoter region of *IRX1* is located in a CpG island (Figure 1b) and hypermethylation of *IRX1* has been found in gastric cancer [15], head and neck squamous carcinoma [16], and other cancer entities [17,18,19]. Functional data indicate that IRX1 acts as an epigenetically regulated tumor suppressor in different types of cancer [15,20].

The chromosomal region 5p15.33, that harbors the *IRX1* gene, has been reported as a susceptibility locus for lung cancer [21,22,23,24]. However, causal genetic and epigenetic variants in this cytoband have not been fully uncovered. In our study, we investigated the epigenetic regulation and the tumor suppressor function of IRX1 in the pathogenesis of lung cancer. Here, we report frequent epigenetic inactivation of *IRX1* in primary lung adenocarcinoma (ADC). Moreover, reduced expression and hypermethylation of *IRX1* was correlated with an impaired prognosis of ADC patients. Gene set enrichment analysis showed a negative correlation of *IRX1* expression with oncogenic hallmarks. Functionally, IRX1 overexpression induced signs of apoptosis including fragmented nuclei and expression of the proapoptotic regulator *BAX*.

## 2. Results

### 2.1. Epigenetic Silencing of IRX1 in Cancer Cells

It has been reported that the chromosomal region 5p15.33 harbors a lung cancer susceptibility locus [21,22,23,24] and we analyzed the epigenetic regulation of *IRX1* that is located in this specific cytoband (Figure 1). Expression of *IRX1* is detected in lung tissue, but also other normal samples including skin, kidney cortex, salivary gland, and breast tissue (Figure S1a) [25]. The *IRX1* gene is localized in an 8.6 kb CpG island containing the whole gene including 1.7 kb upstream of its transcriptional start site (TSS, Figure 1b). We analyzed the promoter methylation of the *IRX1* promoter region of five NSCLC cell lines (A549, A427, HCC15, H322, and H358) and 11 SCLC cell lines (HTB171, HTB175, SCLC21H, SCLC22H, SCLC24H, H82, HTB187, H209, H510, H1092, and H1184) by combined bisulfite restriction analysis (Figure 1c,d). Methylation was determined by a 165 bp *Taq*I restriction fragment in the proximal *IRX1* promoter. We observed partial and full methylation of the diagnostic CpG site for all five NSCLC cell lines, but only for three (HTB171, HTB175, and HTB187) out of 11 SCLC cell lines (Figure 1d). We also found *IRX1* promoter hypermethylation in HeLa cells (Figure 1d).

**Figure 2:**
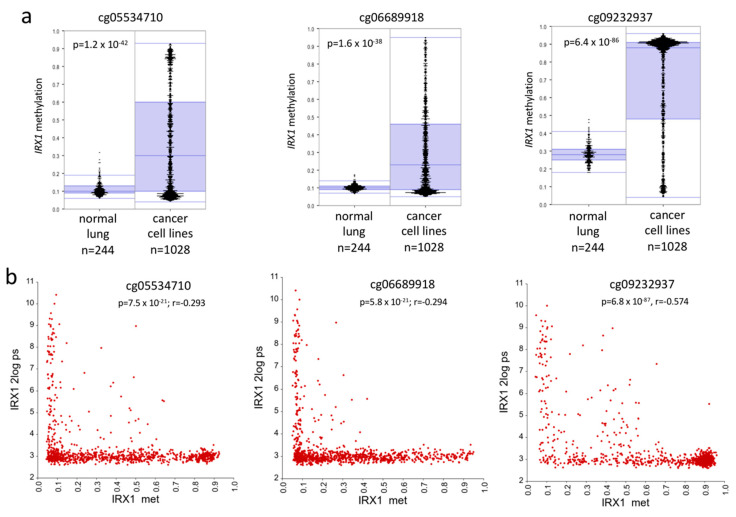
Methylation and expression of *IRX1* in cancer cell lines. (**a**) Methylation level of the *IRX1* promoter at the three CpG sites (cg05534710, cg06689918, and cg09232937) were analyzed by Infinium Human Methylation 450 BeadChip (ilmnhm450K array) through the R2 Genomics Analysis and Visualization Platform [30]. Methylation of 244 normal lung samples (GSE52401) were compared to methylation of 1028 cancer cell lines (GSE68379). *IRX1* methylation levels are plotted in beta-value (1 = 100% methylation) and *p*-value was calculated by one-way ANOVA. (**b**) Correlation analysis of *IRX1* promoter methylation (beta-value) and *IRX1* expression (2log ps). Methylation level of the three CpG sites of cancer cell lines (GSE68379) was plotted for *IRX1* expression (11725275_at) in the corresponding mRNA 1017 of cancer cell lines [31]. Significance of correlation was calculated by the R2 platform. *p* < 0.05 was considered significant.

Downregulation of *IRX1* expression was frequently observed in several cancer cell lines compared to leukemia (Figure S1b) and was also found in NSCLC cell lines [29]. Thus, we analyzed the methylation of three 450K array probes (cg05534710, cg06689918, and cg09232937) in 244 normal lung samples and 1028 cancer cell lines (Figure 2a). A significant *IRX1* hypermethylation was observed for all three CpG sites in cancer cells (Figure 2a). For cg09232937, the median methylation level was higher in both normal lung (30% methylation) and cancer cell lines (90% methylation) compared to the other two CpG sites (normal 10% and cancer cell lines 20–30% median methylation), respectively. Next, we correlated the *IRX1* hypermethylation at these three CpG sites (cg05534710, cg06689918, and cg09232937) with *IRX1* downregulation in these cancer cell lines (Figure 2b). Increased *IRX1* methylation was linked with reduced *IRX1* expression for all three analyzed CpG sites. Cancer cell lines with low *IRX1* methylation (<10%) exhibited the highest level of *IRX1* mRNA levels (Figure 2b). Methylation level at cg09232937 correlated the most significant with the expression of *IRX1*.

### 2.2. Epigenetic Inactivation of IRX1 in Lung Adenocarcinoma Is Correlated with Impaired Prognosis

**Figure 3:**
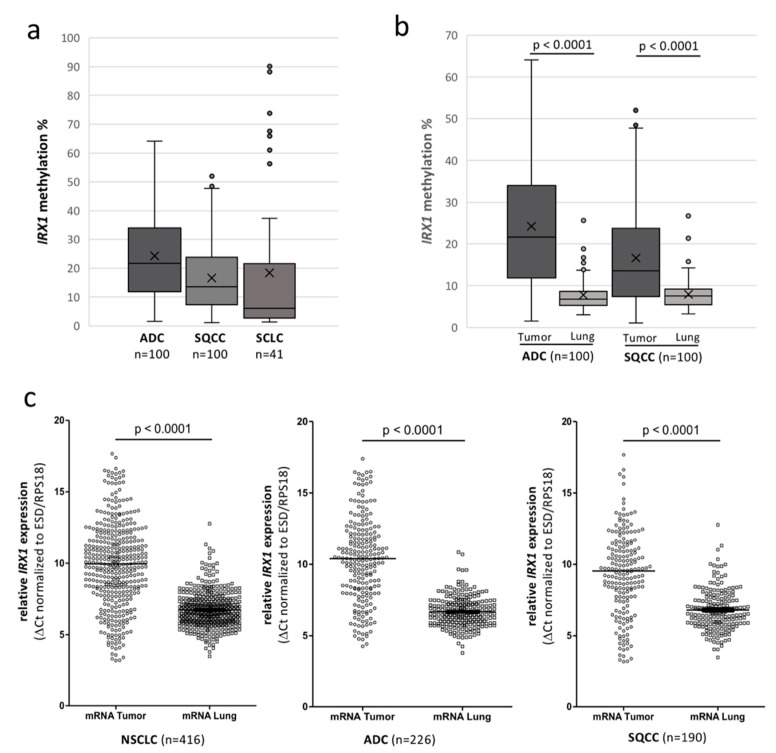
Epigenetic inactivation of *IRX1* in primary lung cancers compared to matching lung. (**a**) Methylation level of the *IRX1* promoter at the nine CpG sites was analyzed in 100 adenocarcinoma (ADC), 100 squamous cell carcinoma (SQCC), and 41 small cell lung cancer (SCLC) samples by bisulfite pyrosequencing. Average CpG methylation levels are depicted by box plot. (**b**) *IRX1* methylation of tumors and corresponding matching lung samples were revealed by pyrosequencing. Average methylation was compared to tumor tissue by box plot and *p*-value was calculated. (**c**) Relative *IRX1* expression in primary tumors and corresponding lung samples. mRNA levels were analyzed by qRTPCR normalized to the housekeeping genes *ESD* and *RPS18* (ΔCt). Note that a higher ΔCt value indicates lower *IRX1* expression. Median expression levels of *IRX1* of NSCLC, ADC, and SQCC and corresponding lung samples are shown and significances (*p*-values) were calculated. *p* < 0.05 was considered significant.

Subsequently, we analyzed the epigenetic inactivation of *IRX1* in 100 lung adenocarcinoma (ADC) and 100 squamous cell carcinoma (SQCC) and the corresponding control samples by bisulfite pyrosequencing and quantitative RT-PCR (Figure 3). Clinicopathological data of all 200 patients with NSCLC are listed in Table S1. Average *IRX1* methylation was determined at nine CpG sites (CpG1-9) flanking the *Taq*I site (CpG3) by sequencing (Figure 1c and Figure 3a). Median methylation levels for all patients are plotted in Figure 3a. In ADC, a higher methylation level (median 22%) was revealed compared to SQCC (median 14%). DNA methylation level of a single CpG site showed a similar pattern for all nine CpG sites in both ADC and SQCC (Figure S2). We also analyzed 41 primary small cell lung cancer (SCLC) samples, that were available from our previous study [4]. In SCLC, a low median *IRX1* methylation (6%) was detected (Figure 3a). However, seven SCLC samples exhibited increased methylation levels (50–90%).

Next, we analyzed the methylation of the nine CpG sites in the corresponding lung tissue of 200 patients with NSCLC and compared the average methylation of ADC and SQCC (Figure 3b and Figure S2). In the matching lung tissue, *IRX1* promoter methylation was found at low levels (median 7% and 8%, respectively) and a significant hypermethylation was found in the corresponding ADC and SQCC (*p* < 0.0001). To analyze the methylation level of *IRX1* in cancer-unrelated tissues, we utilized samples from idiopathic pulmonary artery hypertension (IPAH) patients (Figure S2). Average *IRX1* promoter methylation was at about 5% threshold level, which was lower than the methylation level of normal lung analyzed by 450K bead chip (median of 10% at cg05534710, Figure 2a).

Further, we utilized two independent 450K bead chip data sets of primary ADC (LUAD) and SQCC (LUSQ) samples to verify the epigenetic inactivation of *IRX1* in primary NSCLC by the TCGA Wanderer platform [32]. For ADC, we correlated mean methylation of 463 tumors and 32 normal samples at cg05534710, cg06689918, and cg09232937 and *IRX1* expression (Figure S3a). In ADC tumor samples, a significant hypermethylation of all three CpG sites was found compared to the normal tissues (*p* < 0.001). This increased methylation was inversely correlated with *IRX1* expression for these sites (Figure S3a). For SQCC, methylation and expression data were available for 361 tumor samples and 43 normal lung tissues (Figure S3b). A significant increased mean methylation of SQCC was revealed for all three CpG sites compared to normal samples (*p* < 0.001). Hypermethylation was correlated with a reduced expression for all three CpGs of the *IRX1* promoter (Figure S3b). Thus, we also analyzed the expression of *IRX1* in the available NSCLC RNA samples by qRTPCR (Figure 3c). RNA levels were normalized and reduction of *IRX1* expression is shown as ΔCt values compared to *ESD/RPS18* levels (housekeeping genes). In NSCLC, we found a significant reduction of *IRX1* expression compared to the matching lung tissues (*p* < 0.0001). This significant reduction was also revealed for ADC and SQCC (Figure 3c). However, in ADC, the reduction of median *IRX1* expression was more pronounced than in SQCC. In the corresponding lung disease tissues, this difference was not observed (Figure S3c).

**Figure 4:**
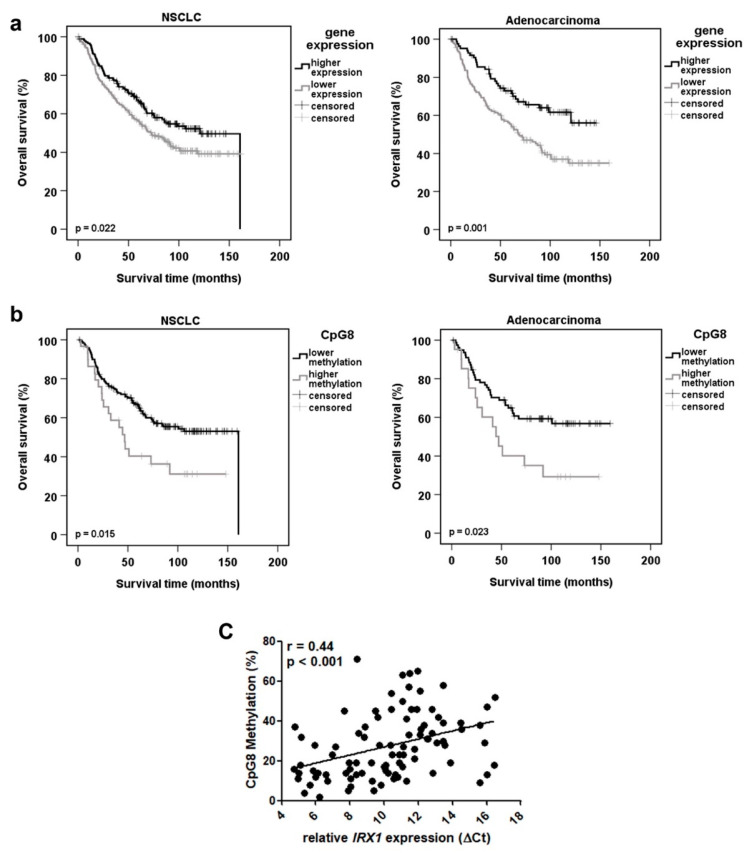
Impaired survival probability of ADC patients is associated epigenetic silencing of *IRX1*. (**a**) To correlate high and low *IRX1* expression with survival probabilities, we performed the Kaplan–Meier estimator for all patients with non-small cell lung cancer (NSCLC) (*n* = 416) and adenocarcinoma (ADC) (*n* = 226) shown in months. (**b**). Survival probability of NSCLC and ADC patients (*n* = 189 and 91, respectively) was correlated with *IRX1* methylation level at CpG site 8 (CpG8). (**c**) Correlation analysis of CpG8 methylation and relative *IRX1* expression (*p* < 0.001). *p* < 0.05 was considered significant.

In order to correlate *IRX1* expression with patient survival probabilities, we performed the Kaplan–Meier estimator and plotted the data in Figure 4 for all patients with NSCLC, but also for ADC and SQCC patients separately (Figure 4a and Figure S4a). Higher *IRX1* expression was associated with a better survival rate of patients with NSCLC (*p* = 0.001; *n* = 226), and this was significantly correlated in ADC (*p* = 0.001; *n* = 226), but not with SQCC (*p* = 0.9; *n* = 190; Figure S4a). By data mining, we confirmed this impaired prognosis for lower *IRX1* expression in two independent data analyses for lung ADC patients (Figure S4b,c), but not for SQCC patients (Figure S4d) [30,33]. Moreover, an association of low *IRX1* expression with advanced NSCLC stages was revealed (Figure S4e).

Next, we correlated *IRX1* methylation levels with survival probabilities of patients with NSCLC (Figure 4b). We observed a significant better survival prognosis for patients with NSCLC harboring a lower CpG8 methylation (*p* = 0.015). A significant correlation was also found for other CpG sites of *IRX1* (Figure S5a). Moreover, lower CpG methylation was also significantly associated with better prognosis for ADC patients (*p* = 0.02; Figure 4b). We also revealed that increased CpG methylation is significantly associated with reduced *IRX1* expression (*p* < 0.001; Figure 4c). Additionally, methylation of cg05534710, cg06689918, and cg09232937 was analyzed by the Kaplan–Meier estimator in 461 patients by the MethSurv tool [34] (Figure S5b). We observed that lower *IRX1* methylation at the CpG sites is correlated with a better prognosis and this trend was significant for cg06689918 and cg09232937 (*p* = 0.03 and 0.02, respectively) (Figure S5a,b).

### 2.3. Downregulation of IRX1 Is Associated with Increased EZH2 and DNMT3A Expression

**Figure 5:**
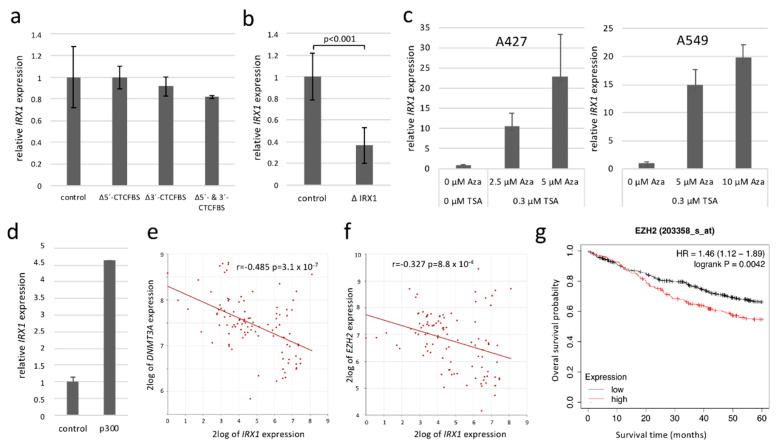
*IRX1* is regulated by DNA methyltransferase and p300 HAT activity. (**a**) Deletion (Δ) of 5′- and 3′-CTCF binding site in U251 cell by CRISPR/Cas9 system. We generated two control clones, three Δ5′-CTCFBS, four Δ3′-CTCFBS, and two Δ5′+3′-CTCFBS clones and analyzed *IRX1* expression for each clone. RNA levels were determined by qRTPCR in triplicates and normalized to *ACTB* level. *IRX1* expression of control clones was set 1 for comparison. (**b**) Deletion of the *IRX1* promoter (ΔIRX1) by CRISPR/Cas9 system. Expression of *IRX1* was analyzed in 13 ΔIRX1 clones by qRTPCR and normalized to *ACTB*. *p*-value was calculated by unpaired t-test. (**c**) A427 and A549 cell lines were treated with the indicated concentration of 5-Aza-2′-deoxycytidine (Aza) and trichostatin A (TSA) for 4 days and RNA was isolated. *IRX1* mRNA levels were analyzed in technical triplicates and normalized to *ACTB*. Then, 0 µM Aza treatment was set 1. (**d**) p300 HAT induced expression of endogenous *IRX1*. HeLa cells were transfected with *IRX1* guide RNA constructs and p300-dCas9 or pcDNA-dCas (control). *IRX1* expression was analyzed by qRTPCR after 1 µM Aza and 0.3 µM TSA treatment for 72 h and normalized to *ACTB* levels. (**e**,**f**) Correlation analysis of *IRX1* and (**e**) *DNMT3A* or (**f**) *EZH2* expression in primary NSCLC samples (GSE33532 data set, *n* = 100). Analysis was performed by R2 [30]. (**g**) Impaired survival probability of adenocarcinoma patients is associated with high *EZH2* expression. Survival probability of ADC patients (*n* = 719) was correlated with *EZH2* expression (low and high) through the Kaplan–Meier plotter [33]. *p* < 0.05 was considered significant.

The *IRX1* gene is located on chromosome 5p15.33 in an 8.6 kb CpG island flanked by the two CTCF binding sites (CTCFBS; Figure 1b). It has been reported that the CTCC-binding protein (CTCF) acts as a tumor suppressor gene by regulating the expression of tumor-related genes [35]. Epigenetic silencing of several tumor suppressor genes is associated with loss of CTCF binding and loss of its function as a chromatin boundary or insulator [36,37]. CTCF binding is inhibited by DNA methylation of its binding site and aberrant methylation of CTCFBS have been reported in human cancer [38,39,40]. Thus, we analyzed the methylation of the 5′- and 3′-CTCBS of the *IRX1* gene by combined bisulfite restriction analysis (Figure S6). We observed a correlation between methylation of the flanking CTCFBS and *IRX1* promoter methylation. In the cancer cell lines SNB18, U343, U251, U118, and T47D on CTCFBS and the *IRX1* promoter are unmethylated (Figure S6). In contrast, the other cell lines exhibited hypermethylation of both CTCFBS and of the *IRX1* promoter. To analyze the impact of the CTCF binding sites flanking the *IRX1* CpG island, we utilized the CRISPR/Cas9 system to delete the 5′- and/or 3′-CTCFBS in the U251 cell line which expresses *IRX1* (Figure 5a).

We generated two control clones, three Δ5′-CTCFBS, four Δ3′-CTCFBS, and two Δ5′+3′-CTCFBS clones and analyzed *IRX1* expression (Figure 5a). Deletion of CTCFBS was verified by PCR and conventional sequencing for all clones. *IRX1* levels were determined by qRTPCR and plotted relative to the control clones. However, deletion of CTCFBS (5′ or/and 3′) showed no significant effect on *IRX1* expression (Figure 5a). We also generated a deletion of the *IRX1* promoter (ΔIRX1) by CRISPR/Cas9 system (Figure 5b). Expression of *IRX1* was investigated in 13 ΔIRX1 clones and we observed a significant reduction in *IRX1* expression compared to controls (2.7-fold median reduction; *p* < 0.0001).

To analyze the reactivation of *IRX1* expression in lung cancer cell lines, we treated A427 and A549 cells with an inhibitor of DNMT (Aza/5-Aza-2′-deoxytidine) and an inhibitor of HDAC (TSA/trichostatin A) (Figure 5c). Both cell lines were treated with increasing concentration of Aza and 0.3 µM TSA. In A427 and A459 lung cancer cell lines, we observed a dosage dependent induction of *IRX1* expression by the DNMT inhibitor (20-fold induction by 5 or 10 µM Aza, respectively) (Figure 5c). Subsequently we investigated the effect of epigenetic editing by promoter specific recruitment (CRISPR/dCas9 system) of histone acetyltransferase p300 HAT to the *IRX1* promoter in HeLa cells, that harbor hypermethylated *IRX1* promoter (Figure 1d). In this tethering experiment, we revealed that recruitment of p300 HAT to the *IRX1* promoter induced the expression of *IRX1* 4.5-fold compared to the control (Figure 5d). Next, we analyzed the binding of transcriptional regulators to the promoter region of *IRX1* by ChIP-seq in the ENCODE data set (Figure S7a). This data revealed that EZH2 binds several positions of the *IRX1* promoter region. In lung cancer, overexpression of DNMTs and *EZH2* has been reported [3,41,42]. Interestingly, we also observed an inverse correlation between *IRX1* expression and *DNMT3A* or *EZH2* levels in primary NSCLC samples (Figure 5e,f, respectively). This significant reduction of *IRX1* level associated with higher DNMT3A or EZH2 expression was confirmed in three independent NSCLC data sets (Figure S7b). In order to correlate *EZH2* expression with patient survival probabilities, we performed the Kaplan­–Meier estimator (Figure 5g). Higher *EZH2* level was associated with an impaired survival rate of patients with ADC (*p* = 0.0042; *n* = 719).

By gene ontology analysis, *IRX1* expression was also linked to the GO term cell cycle in four NSCLC data sets (Table 1). We also correlated *IRX1* expression by gene set enrichment analysis (GSEA) by the Genomics Analysis and Visualization Platform R2 (Table 1). *IRX1* expression was significantly, negatively correlated with hallmarks E2F- and Myc-targets, mTORC1 signaling, and G2M checkpoint in these microarrays (Table 1). Additionally, in the data set GSE19804 [43] *IRX1* expression was also negatively associated with hallmark EMT (*p* = 0.00063) and positively with myogenesis (*p* = 0.0013). Interestingly, in the TCGA-LUAD data set, *IRX1* expression was also significantly correlated with the hallmark p53-pathway (Table 1).

### 2.4. Overexpression of IRX1 Is Associated with Fragmented Nuclei and Induction of BAX

**Figure 6:**
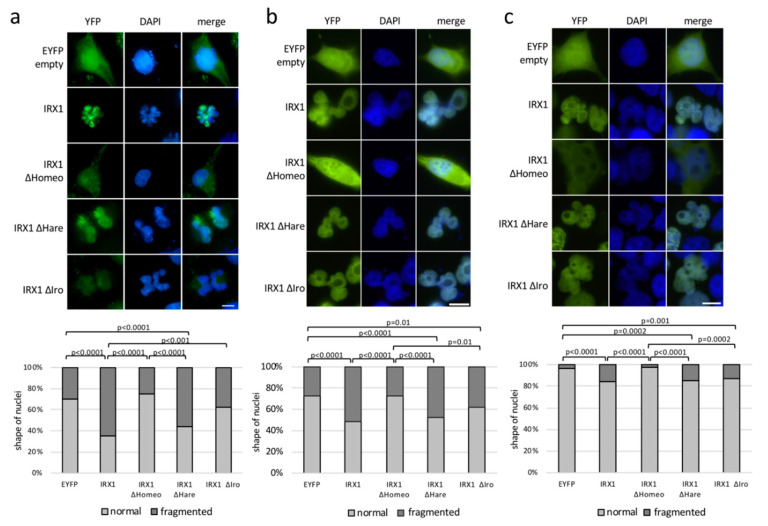
Nuclear localization of IRX1 and irregular shape of IRX1 expressing nuclei. IRX1 was fused to EYFP and transfected in A549 (**a**) HeLa (**b**) and HEK293T (**c**) cells. Localization of IRX1 in the nucleus was analyzed by DAPI co-staining and fluorescent microscopy. IRX1 deletion construct of homeobox (ΔHomeo), HARE-HTH domain (ΔHare), and IRO box (ΔIro) were generated and transfected. The nuclear shape of transfected cells was analyzed after 24 h in A549 (80–160 cells analyzed), HeLa (200–350 cells), and HEK293 (150–300 cells). Normal nuclei exhibited a round shape and fragmented nuclei showed an irregular, lobed shape. Significance was calculated by Fisher’s exact test. White bar represents the length standard of 5 µM.

To investigate the cellular function of IRX1, we fused its ORF with an enhanced yellow fluorescent protein (EYFP) and transfected this construct in A549 lung cancer cells (Figure 6). EYFP tagged IRX1 was localized in the nucleus in A549 cells and we noted that IRX1-expressing cells had irregular shapes and fragmented nuclei (Figure 6a). Additionally, we utilized HeLa cancer cells (Figure 6b) and HEK293T cells (Figure 6c) to verify the shape of nuclei upon IRX1 transfection. In both cell lines, we observed a significant accumulation of fragmented nuclei in the IRX1-expressing cells compared to control cells (EYFP). Subsequently, we deleted specific domains of IRX1 (Figure 1), including the homeobox (ΔHomeo), the HARE-HTH domain (ΔHare), and the IRO box (ΔIro) by site directed mutagenesis. We transfected these IRX1-deletion constructs in A549 (Figure 6a), HeLa (Figure 6b), and HEK293 cells (Figure 6c) and analyzed the localization of these proteins.

**Figure 7:**
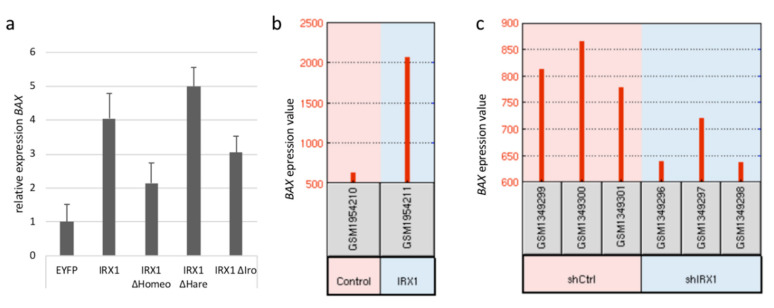
*IRX1* expression is associated with expression of the apoptotic regulator BAX. (**a**) IRX1 wt and IRX1 deletion construct of homeobox (ΔHomeo), HARE-HTH domain (ΔHare), and IRO box (ΔIro) were transfected in HeLa cells. RNA was isolated after 48 h and *BAX* mRNA levels were analyzed in technical triplicates and normalized to *ACTB*. *BAX* expression was plotted relative to EYFP-control transfected HeLa cells (set = 1). (**b**) *BAX* expression values (208478_s_at) were analyzed in pcDNA3.1-GFP (control) and pcDNA3.1-IRX1-GFP transfected HEK293T cells in the data set GSE75376 by GEO2R [46,47]. (**c**) Expression of *BAX* (NM_004324) was analyzed in 143B cells after *IRX1* (shIRX1) and control (shCtrl) knock down in technical triplicates in the data set GSE56255 by GEO2R [47,48].

Interestingly, we observed that the IRX1-ΔHomeo protein exhibited a similar localization as control EYFP transfected cells and that the induction of fragmented nuclei was significantly associated with the presence of the IRX1 homeobox (Figure 6). This indicates that the homeobox is an important functional domain of IRX1 and responsible for the nuclear localization. ΔHomeo- and ΔIro-IRX1 constructs showed a nuclear localization and also induced fragmented nuclei in all three cell lines. However, in A549 cells, ΔIro-IRX1 induced significantly fewer fragmented nuclei compared to full length IRX1 (Figure 6a). Subsequently, we analyzed the effect of *IRX1* expression on the apoptosis regulator *Bcl2-associated X (BAX)* (Figure 7). Therefore, we transfected IRX1 in cancer cells and analyzed the mRNA levels of *BAX* (Figure 7a). Compared to the control cells, we observed a 4-fold induction of *BAX* in the *IRX1* expressing HeLa cells. However, in IRX1-ΔHomeo overexpressing cells, *BAX* induction was reduced to only 2-fold. *BAX* mRNA levels, in cells transfected with the deletion constructs of the HARE-HTH domain (ΔHare) or the IRO box (ΔIro), were comparable to full length IRX1 (Figure 7a).

We also analyzed the expression values of *BAX* in IRX1-expressing HEK293T cells by GEO2R [46,47]. A 1.7-fold induction of *IRX1* was revealed in the data set GSE75376 (Figure S8). *IRX1* expression was associated with a 3.2-fold induction of *BAX* values compared to control transfected HEK293T cells (Figure 7b). Additionally, the effect of *IRX1*-knock down (shIRX) was investigated in 143B osteosarcoma cells [48] (Figure 7c). Compared to control-knock down (shCtrl), *IRX1* levels were 2-fold reduced in the shIRX1 cells (Figure S8). Knock down of *IRX1* (shIRX1) resulted in reduced *BAX* levels compared to 143B-shCtrl cells. This data indicates that *IRX1* levels are linked with the expression of the apoptosis regulator BAX.

## 3. Discussion

In our study, we wanted to understand the regulation of *IRX1* in lung cancer and its molecular contribution to tumorigenesis. *IRX1* is localized on cytoband 5p15.33 and it has been reported that this region harbors a susceptibility locus for lung cancer [21,22,23]. Here, we show that *IRX1* is expressed in normal lung tissue (Figure S1a) and downregulated in NSCLC tumors (Figure 3). Additionally, we revealed that the reduced expression of *IRX1* in cancer is linked to its promoter hypermethylation (Figure 2 and Figure 4c). In normal lung, the *IRX1* promoter exhibits low methylation (<10%) and this result has been found for two CpG sites by 450K bead chip (Figure 2) and for nine CpGs by bisulfite pyrosequencing (Figure 3b). The third 450K CpG probe (cg09232937) exhibits, in general, a higher methylation level in normal lung, but also in cancer cell lines (Figure 2 and Figure S3). It would be interesting to verify the methylation level of this specific CpG site by conventional bisulfite sequencing. However, this genomic region is very CpG- and GC-rich and therefore difficult to sequence (Figure 1c).

For gastric cancer, an SNP (rs11134044) has been associated with differential expression of *IRX1* and correlated with cancer risk by a genome-wide association study (GWAS) [49]. Interestingly, this SNP is located 700 kb downstream of the *IRX1* gene and this region could harbor an *IRX1* enhancer. A lung cancer associated risk of the biomarker rs11134044 has been not reported by GWAS. Regulation of gene activity though distal enhancers are often linked to genomic CTCF binding sites (CTCFBS) and methylation of these sites block CTCF binding [50,51]. Epigenetic alteration or mutation of CTCFBS have been associated with deregulation of tumor suppressor genes [36,37,38,52]. Here, we observed a link between methylation of the CTCFBS flanking the 5′- and 3′-shore of the *IRX1* CpG island and the methylation of the *IRX1* promoter (Figure S6). Therefore, we deleted the two CTCF consensus sites by CRISPR/Cas9 technology and analyzed the expression of *IRX1*. Deletion of both CTCFBS resulted only in a mild reduction of *IRX1* mRNA levels, compared to the proximal *IRX1* promoter deletion (Figure 5).

In lung cancer, overexpression of DNMTs and *EZH2* has been reported [3,41,42]. EZH2 is histone H3 lysine 27 methyltransferase and the catalytic subunit of the transcriptional repressive complex of PRC2. ChIP-seq data revealed frequent binding of EZH2 to a 3 kb sequence flanking the transcriptional start site of the *IRX1* gene (Figure S7a). EHZ2 directly controls DNA methylation through interaction with DNMTs [53]. This data suggests that *IRX1* is an EZH2-target promoter and EZH2 may act as a recruitment platform for de novo methyltransferase DNMT3A [53]. Subsequently, we analyzed the correlation of *IRX1* expression and levels of *DNMT3A* and *EZH2* (Figure 5e,f and Figure S7b). Here, we observed an inverse correlation of *IRX1* and *DNMT3A* or *EZH2* expression in primary lung cancers suggesting a causal relation between aberrant expression of epigenetic modifiers and *IRX1* silencing. We also found that high *EZH2* level are associated with an impaired prognosis of patients with lung adenocarcinomas (Figure 5g). Moreover, we recruited the p300 HAT to the *IRX1* promoter in HeLa cells (Figure 5d) and observed a 4.5-fold induction of endogenous *IRX*1 level. Interestingly, we observed that *IRX1* was already downregulated in cancer cell lines that exhibited only low methylation levels (Figure 2b). It is tempting to speculated that repressive histone marks (deacetylation and methylation) and precedes DNA hypermethylation of the *IRX1* promoter as previously reported for the epigenetic downregulation of RASSF1A tumor suppressor gene [54]. Additionally, inhibition of DNMT activity by 5-Aza-2′-deoxycytidine reactivated the expression of *IRX1* in two lung cancer cell lines (Figure 5c). It will be interesting to analyze the exact role of DNMT3A and EZH2 in the epigenetic regulation of *IRX1* in lung carcinogenesis.

Since there is evidence that epigenetic control plays a significant role in the pathogenesis of another lung disease (IPAH) [55], we also analyzed *IRX1* hypermethylation in IPAH samples. However, low methylation level (<10%) were observed in IPAH tissues (Figure S2) and this indicates that DNA hypermethylation of *IRX1* is rather associated with lung cancer than with IPAH. In primary SCLC samples *IRX1* exhibits also low median methylation (6%), but some SCLC tissues and cell lines showed high methylation levels (>50%; Figure 1d and Figure 3a). Thus, aberrant *IRX1* methylation in SCLC is rather heterogenous compared to NSCLC samples. In adenocarcinoma (ADC), we observed frequent increased methylation of the *IRX1* promoter and reduced RNA levels (Figure 3). This epigenetic inactivation of *IRX1* was significantly correlated with a reduced survival probability on RNA and DNA methylation levels (Figure 4). These results that low expression and high methylation of *IRX1* are associated with an impaired prognosis of ADC patients were also verified in independent data sets (Figures S4 and S5, respectively). Guo et al. analyzed the prognostic value of *IRX1* methylation in squamous cell carcinoma of the lung (LUSQ) and reported that methylation of two CpG sites are significantly associated with the prognosis of LUSQ patients [18]. In our experiments, we also observed epigenetic inactivation of *IRX1* in SQCC, but the reduced *IRX1* level and higher *IRX1* methylation were less pronounced compared to ADC (Figure 3). Therefore, we could not reveal such a prognostic value of aberrant *IRX1* expression or methylation in SQCC (Figure S4). Yu et al. observed that *IRX1* regulates the epithelial differentiation of lung alveolar type II cells [10]. In patients, adenocarcinomas often stain positively with antibodies to markers of the alveolar type II cells and this led to hypothesis that alveolar type II cells could be cells of origin in ADC [56]. Thus, it will be interesting to decipher the functional differences of IRX1 in pathogenesis of ADC compared to SQCC and to analyze the expression of *IRX1* in progenitor cells of the lung.

Hypermethylation of *IRX1* has been found in gastric cancer [15] or head and neck squamous carcinoma [16]. In gastric cancer, IRX1 acts a tumor suppressor through downregulation of BDKRB2, FGF7, and HIST2H2BE expression and inhibiting angiogenesis and cell proliferation [15]. Consequently, restoring *IRX1* expression in two gastric cancer cell lines inhibited growth, invasion, and tumorigenesis in vitro and in vivo [15]. Here, we report that *IRX1* expression is negatively correlated with the hallmark EMT a lung cancer data set (Table 1). In head and neck squamous carcinoma cells, overexpression of IRX1 resulted in decreased mitotic activity and increased apoptosis [57]. In our study, we analyzed the localization of IRX1 and we found that overexpression of IRX1 induced fragment nuclei in lung adenocarcinoma cells (Figure 6a). Additionally, we revealed that *IRX1* induced the expression of the proapoptotic regulator *BAX* that is also a p53 target gene (Figure 7). This data suggests that *IRX1* is positively associated with the p53 pathway and this has been confirmed by GSEA in the TCGA-LUAD data set (Table 1). Kühn et al. demonstrated that *IRX1* overexpression in HEK293 cells is positively associated with GO term apoptosis [46] and in this data set we confirmed induction of *BAX* through *IRX1* (Figure 7b). Interestingly, the induction of *BAX* and fragmented nuclei by *IRX1* relied on the presence of its homeobox (Figure 6 and Figure 7a). Homeobox domains are characteristic protein folds that bind DNA and regulate the expression of target genes during development and differentiation. Jung et al. analyzed the function of *IRX1* in the development of zebrafish and they demonstrated that *IRX1* acts a tumor suppressor in multiple organs by regulating the cell cycle progression [20]. Here, we revealed by GSEA that *IRX1* expression is negatively correlated with E2F- and Myc-targets in several lung cancer data sets (Table 1). In general, E2F- and Myc-targets are pro-proliferative factors that induce cell cycle progression and both factors are inhibited by tumor suppressors.

In summary, our data suggest that *IRX1* acts as tumor suppressor gene in the pathogenesis of lung adenocarcinoma and epigenetic silencing of *IRX1* is associated with advanced stage and impaired prognosis of ADC patients.

## 4. Materials and Methods

### 4.1. Cell lines, Lung Cancer Tissues, and Controls

NSCLC cell lines (A427, A549, H322, H358, and HCC15) and SCLC cell lines (HTB171, HTB175, SLC21H, SCLC22H, SCLC24H, H82, HTB187, H209, H510, H1092, and H1184) were described previously [4,58]. Patient samples were provided by Lungbiobank Heidelberg, a member of the Biomaterialbank Heidelberg (BMBH) and the Biobank Platform of the German Centre For Lung Research (DZL) [59] or characterized previously [3,4]. Clinicopathological data of patients with NSCLC are listed in Table S1. All patients signed informed consent at initial clinical investigation. The study was approved by local ethic committees.

### 4.2. Cell Culture and Treatment of Cell Lines

Cell lines were grown in appropriate medium (DMEM/Dulbecco’s modified Eagle’s medium, RPMI/Roswell Park Memorial Institute) supplemented with 10% fetal calf serum and 1% penicillin/streptomycin under cell culture conditions (37 °C, 5% CO_2_). For 5-Aza-2′-deoxycytidine (Aza, Sigma, Darmstadt, Germany) and trichostatin A (TSA, Sigma, Darmstadt, Germany) treatment cells were split to 10% density. Then, 2.5, 5, or 10 µM Aza and 0.3 µM TSA were added with fresh medium on 4 consecutive days before RNA isolation. Cell lines were transfected for indicated time points using polyethylenimine (PEI, 4.9 mM, Sigma, Darmstadt, Germany) for HEK293T, Turbofect (Thermo Fisher Scientific, Waltham, MA, USA) for A549, A427, and HeLa or X-tremeGENE HP (Roche, Basel, Switzerland) for U251 according to the manufacturer’s protocol with 4 μg DNA (6 well plates) or 10 μg DNA (10-cm dishes).

### 4.3. DNA Isolation and Methylation Analysis

Primers for bisulfite treated DNA were designed to bind only fully converted DNA and amplify *IRX1* promoter region. DNA methylation of the *IRX1* promoter was analyzed by combined bisulfite restriction analysis (CoBRA) or pyrosequencing [60]. DNA isolation was done after proteinase K (Thermo Fisher Scientific, Waltham, MA, USA) digest, extracted with phenol/chloroform and concentrations were determined by UV-photospectrometery. For cell line COBRA methylation analysis, 2 µg genomic DNA was bisulfite treated (12 µL 0.1 M hydroquinone, 208 µL 1.9 M sodium metabisulfite, and pH 5.5 with NaOH) and incubated over night at 50 °C. DNA was purified using MSB Spin PCRapace (STRATEC Molecular), eluted in 50 µL H_2_O, and followed by 10 min incubation with 5 µL 3 M NaOH at 37 °C. DNA was then precipitated with 100% ethanol and 7.5 M ammonium acetate and resolved in 1 × TE buffer. For patient CoBRA methylation analysis, 250 ng genomic DNA was bisulfite treated using EZ DNA methylation-lightning (Zymo Research, Freiburg, Germany). The CoBRA PCR was performed semi-nested with upper primer 1 TAAATTYGGTGTTAGATAGTTGTAAATT and lower primer 1 AAACTTTCAAACACAATTTAAAATTA, and upper primer 1 and lower primer 2 TAATACTTTAATTAACATCCCCTTAA. The semi-nested PCR product for *IRX1* is 272 bp (*Taq*I digest products of 33, 74, and 165 bp). The subsequent PCR product was digested with 5 U of *Taq*I (Thermo Fisher Scientific, Waltham, MA, USA) 1 h at 65 °C and resolved on 2% TBE gel together with mock control and DNA ladder. In vitro methylation (ivm) of genomic DNA was performed using CpG Methyltransferase *M.Sss*I (NEB, Frankfurt, Germany) according to manufacturer’s protocol. Pyrosequencing was performed according to manufacturer’s protocol with PyroMark Q24 System (Qiagen, Hilden, Germany), biotinylated lower primer 2, and sequencing primer GGTTTTYGGGTTTATATTT.

### 4.4. Patient Biospecimen

This biomarker study was conducted in accordance with the Reporting Recommendations for Tumor Marker Prognostic Studies [61]. Tissue samples were provided by the Lung Biobank Heidelberg, a member of the accredited Tissue Bank of the National Center for Tumor Diseases (NCT) Heidelberg, the Biomaterial Bank Heidelberg, and the Biobank platform of the German Center for Lung Research (DZL). The use of biomaterial and data for this study was approved by the local ethics committee of the Medical Faculty Heidelberg (S-270/2001). All patients included in the study signed an informed consent and the study performed according to the principles set out in the WMA Declaration of Helsinki. Tumor and matched distant (>5 cm) normal lung tissue samples from patients with NSCLC who underwent therapy-naive resection for primary lung cancer at Thoraxklinik at University Hospital Heidelberg, Germany were collected between 2006 and 2011. Tissues were snap-frozen within 30 min after resection and stored at −80 °C until the time of analysis. More detailed information is described elsewhere [59].

Human lung tissue explanted from patients with PAH was obtained during lung transplantation. Similarly, donor lung samples obtained from downsized healthy lung tissues to serve as a reference to PAH samples. The study protocol for tissue donation was approved by the Ethics Committee (Ethik Kommission am Fachbereich Humanmedizin der Justus Liebig Universität Giessen) of the University Hospital of Giessen, Germany, in accordance with national law and Good Clinical Practice/International Conference on Harmonisation guidelines. Written informed consent was obtained from individual patients or the patient’s next of kin (AZ 31/93).

### 4.5. RNA Expression Analysis

RNA from human cell culture was isolated using Isol-RNA lysis procedure (Trizol, Thermo Fisher Scientific, Waltham, MA, USA). RNA was *DNase* (Thermo Fisher Scientific, Waltham, MA, USA) treated and then reversely transcribed by MMLV (Promega, Madison, WI, USA). Quantitative RT–PCR was performed in triplicate with SYBR select (Thermo Fisher Scientific, Waltham, MA, USA) using Rotor-Gene 3000 (Qiagen, Hilden, Germany). The following primers were used for qPCR: IRX1RTF1 (GCGGATCTCAGCCTCTTCTCG), IRX1RTL4 (GGGGTCCCCGTATTGGAACT), BAXRTR (CGCGGTGGTGGGGGTGAGG), BAXRTF (AACTGGGGCCGGGTTGTCGC), BACTRTFW (CCTTCCTTCCTGGGCATGGAGTC), BACTRTRV (CGGAGTACTTGCGCTCAGGAGGA).

For RNA isolation from patient tumor tissue, a tumor content of ≥50% was the minimum prerequisite. A total of 10–15 tumor cryosections (10–15 µM) from each patient were sliced, and the first as well as the last section of the series was stained with H&E. A lung pathologist determined the proportion of viable tumor cells, stromal cells, healthy lung cells, and necrotic areas. Total RNA was isolated from patient tissue using an AllPrep DNA/RNA/miRNA Universal kit (Qiagen, Hilden, Germany) according to the manufacturer’s instructions. A RNeasy Mini kit (Qiagen, Hilden, Germany) was used to isolate RNA from the cell lines. Afterwards, the quality of total RNA was assessed by utilizing an Agilent 2100 Bioanalyser and an Agilent RNA 6000 Nano kit (Agilent Technologies, Boeblingen, Germany). With the Transcriptor First Strand cDNA Synthesis kit (Roche, Basel, Switzerland), total RNA was transcribed to complementary DNA and used for quantitative polymerase chain reaction (qPCR). A complete description of the procedure is provided elsewhere [62]. For gene expression analyses of patient tissues, volumes of 5 µL cDNA (corresponding to 5 ng of isolated total RNA) were utilized for qPCR with the LightCycler480 (Roche, Basel, Switzerland) in 384-well plates according to the Minimum Information for Publication of qPCR Experiments (MIQE) guidelines [63]. Universal probe library (UPL) assay (Roche, Basel, Switzerland) was used as the amplification and detection system. Gene-specific primers (TIB Molbiol, Berlin, Germany) were combined with the primaQuant 2×qPCR Probe-MasterMix (Steinbrenner Laborsysteme, Wiesenbach, Germany). Threshold cycle (Ct) values were evaluated with the LightCycler480^®^ software release 1.5 and the 2nd derivative maximum method (Roche, Basel, Switzerland). For the comparison of gene expression in tumor and non-malignant samples, the relative expression of the genes (normalized to the housekeepers *Esterase D* (*ESD*) and *Ribosomal Protein S18* (*RPS1*8)) was calculated (ΔCt values). The following primers and UPL were used for the detection of *IRX1*: IRX1 forward (UPL#66, GGGCACTCAATGGAGACAA), IRX1 reverse (UPL#66, CTGGGGACGAGGTCTCTCT), ESD forward (UPL#50, TCAGTCTGCTTCAGAACATGG), ESD reverse (UPL#50, CCTTTAATATTGCAGCCACGA), RPS18 forward (UPL#46, CTTCCACAGGAGGCCTACAC), RPS18 reverse (UPL#46, CGCAAAATATGCTGGAACTTT).

### 4.6. Plasmids

The full length *IRX1* coding sequence (BC166635) was obtained from BioCat (Heidelberg, Germany) and cloned into pEYFP (Clontech, Saint-Germain-en-Laye, France). IRX1 domains were deleted by site-directed mutagenesis (QuikChange Lightning, Agilent, Boeblingen, Germany) and verified by sequencing and fluorescence microscopy. Oligos for mutagenesis were ∆Homeobox: CAGCACGCTCAAGGCCCTCAAGAAGGAGAACAA and TTGTTCTCCTTCTTGAGGGCCTTGAGCGTGCTG, ∆HARE-HTH: GGCGCCTCAAGAAGAAGGCCGAGGCTCC and GGAGCCTCGGCCTTCTTCTTGAGGCGCC and ∆Irobox: GGCGCACCGTCGCCGTGCGGCG and CGCCGCACGGCGACGGTGCGCC.

### 4.7. Genetic and Epigenetic Editing

Cas9 vector px549 (SpCas9-2A-PuroV2.0, Addgene, Watertown, USA) was obtained from Lienhard Schmitz (Giessen, Germany) and adapted for epigenetic editing. The *IRX1* and CTCFBS targeting guide RNAs were cloned into px549 under U6 promoter. Single oligos for *IRX1* RNA guides for promoter deletion are #1 CGCTCCTCCCTAGACCCCTCG and #2 CCGCAGTCGGCGCGCGAT and are positioned relative to TSS at −61 #1 and −185 #2, respectively. Oligos for RNA guides for the deletion of 5′-CTCFBS motif (hg19: chr5:3,594,998-3,595,024) are CGCCGCCGCCAGACCGGCAA and GAGAAGAGGGACGCGGGACT and for deletion of 3′-CTCFBS motif (hg19: chr5:3,603,194-3,603,218) are AGTCGGAATGGACGTGCTCC and TCAAGGCCGCATTCCGCTTT. We transfected U251 cells with the Cas9 ΔIRX1- or ΔCTCFBS-guide constructs and selected for positive clones by puromycin (1 µg/mL) for three days. Clones were expanded and the knockout was verified by PCR based amplification of the flanking genomic region and conventional sequencing.

For epigenetic editing, px549 was adapted by deletion of Cas9. *IRX1* RNA guides for promotor reporter assay are #1, #3 CGCGACCGGCCTCCATC (−166), #5 TTGCCGGTCTGGCGGCGGCGC (-1201), and #6 GAGAAGAGGGACGCGGGACT (positioned -1242). p300 (pcDNA-dCas9-p300 Core, Addgene, Watertown, USA) was used as epigenetic modifier upon co-transfection in HeLa cells [64].

### 4.8. Fluorescent Microscopy

For morphological analysis, cells were seeded on glass slides and transfected the following day. Cells were fixed with 3.7% formaldehyde at according time points, permeabilized using TritonX, stained with DAPI (0.1 μg/mL in PBS, Sigma, Darmstadt, Germany), embedded in anti-fading with Mowiol (Sigma, Darmstadt, Germany) and analyzed with Axio Observer Z1 (Zeiss, Oberkochen, Germany) under ×63 magnification and Velocity Software (Perkin Elmer, Waltham, MA, USA).

### 4.9. Data and Statistical Analysis

Gene expression analysis, promoter methylation correlation, and Kaplan–Meier calculations were performed using Genomics Analysis and Visualization Platforms: R2 [30], Wanderer [32], GTEx [25], KM Plotter [33], GEO2R [47] and MethSurv [34].

## 5. Conclusions

Lung cancer incidences are growing and therefore the requirement for novel treatment options and detection methods are rising. In our work, we identified *Iroquois homeobox 1* (IRX1) from the lung cancer susceptibility locus 5p15.33, as an epigenetically silenced target gene. We also analyzed the regulation of *IRX1* as a potential target for lung cancer diagnosis and its reactivation as a novel therapeutic option for epigenetic editing. We are showing that inactivation of *IRX1* is linked to aberrant expression of *DNMT3A* and *EZH2* and that inhibition of DNMT and HDAC induces *IRX1* expression. Additionally, we validated the *IRX1* promoter as a target for epigenetic editing. We are also showing that *IRX1* expression is negatively associated with oncogenic hallmarks by GSEA and that IRX1 expression induces signs of apoptosis in cancer cells, including fragmented nuclei and *BAX* levels. In different datasets, we could validate that epigenetic silencing of *IRX1* is clinically correlated with impaired survival and with progressed disease state of lung adenocarcinoma patients. Thus, loss of *IRX1* expression by its promoter hypermethylation can serve as a diagnostic and prognostic lung cancer biomarker.

## Figures and Tables

**Table 1:** Significances of hallmarks correlated with *IRX1* expression in NSCLC data sets by gene set enrichment analysis (GSEA).

Hallmarks ^1^	GSE33532 [44]	GSE19804 [43]	TCGALUAD	GSE63074 [45]
E2F_targets ^2^	1.1 × 10^−6^	1.0 × 10^−15^	1.7 × 10^−19^	6.2 × 10^−9^
Myc_targets_V1 ^2^	1.1 × 10^−6^	5.2 × 10^−3^	-	-
mTORC1_signaling ^2^	9.2 × 10^−5^	1.6 × 10^−4^	3.6 × 10^−3^	9.0 × 10^−3^
G2M_checkpoint ^2^	5.6 × 10^−4^	1.6 × 10^−11^	1.3 × 10^−10^	1.4 × 10^−4^
EMT ^2^	-	6.3 × 10^−4^	-	-
myogenesis ^3^	-	1.3 × 10^−3^	-	-
p53_pathway ^3^	-	-	3.6 × 10^−3^	-

^1^ geneset_broad_2019_h_hallmark, ^2^ negative correlation, ^3^ positive correlation.

## Supplementary Materials

The following are available online at https://www.mdpi.com/2072-6694/12/12/3528/s1, Figure S1: Expression of *IRX1* in normal tissues and cancer cell lines; Figure S2: *IRX1* promotor methylation of 9 analyzed CpG sites in NSCLC and IPAH patients; Figure S3: Methylation and expression of *IRX1* in ADC (LUAD) and SQCC (LUSQ); Figure S4: Impaired survival probability of adenocarcinoma patients and advanced tumor stages are associated with low *IRX1* expression; Figure S5: Impaired survival probability of NSCLC patients is associated higher *IRX1* methylation; Figure S6: Methylation analysis of the *IRX1* 5′- and 3′-CTCF binding sites in cancer cell lines; Figure S7: Regulation of *IRX1* by EZH2 and DNMT3A; Figure S8: *IRX1* expression after IRX1 transfection in HEK293T cells and *IRX1* knock down in osteosarcoma 143B cells; Table S1: Summary of clinicopathological data of NSCLC patients.
