# Supplementary material for: Epigenetic Inactivation of the Tumor Suppressor IRX1 Occurs Frequently in Lung Adenocarcinoma and Its Silencing Is Associated with Impaired Prognosis
Source: Cancers (Basel). 2020 Nov 26;12(12):3528. doi: 10.3390/cancers12123528 (PMC7760495; doi:10.3390/cancers12123528)
Supplement: Supplementary file 1 [file cancers-12-03528-s001.pdf]

cancers

MDPI

Supplementary Materials

Epigenetic Inactivation of the Tumor Suppressor *IRX1* Occurs Frequently in Lung Adenocarcinoma and Its Silencing Is Associated with Impaired Prognosis

Miriam M. Küster, Marc A. Schneider, Antje M. Richter, Sarah Richtmann, Hauke Winter, Mark Kriegsmann, Soni S. Pullamsetti, Thorsten Stiewe, Rajkumar Savai, Thomas Muley and Reinhard H. Dammann

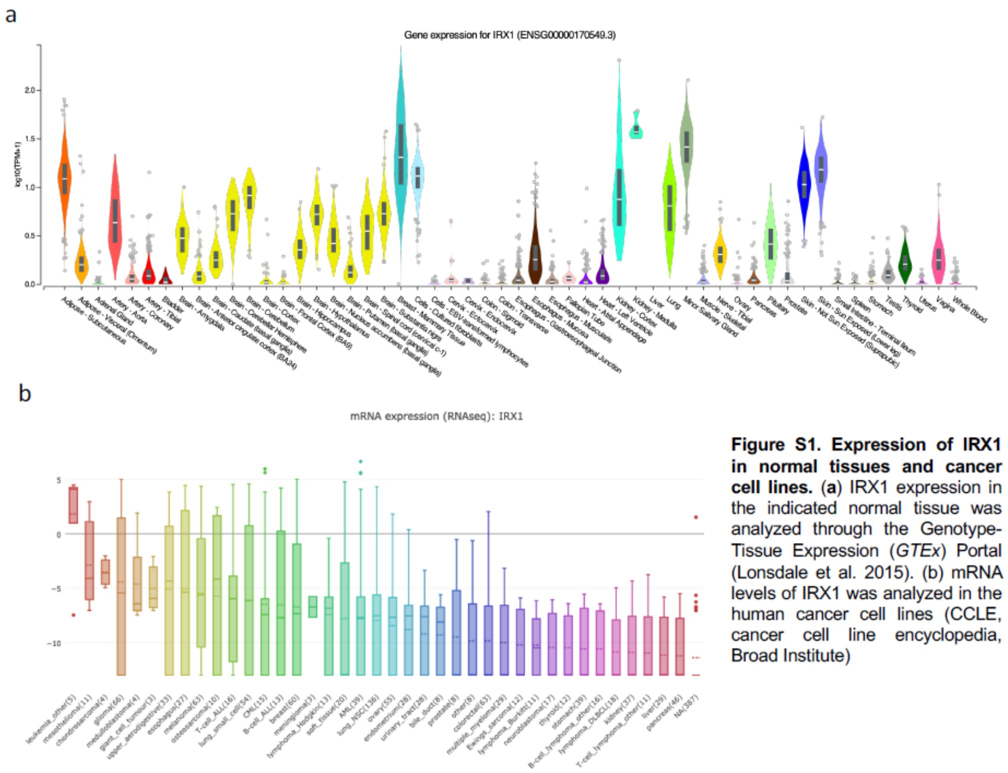

Figure S1. Expression of IRX1 in normal tissues and cancer cell lines. (a) IRX1 expression in the indicated normal tissue was analyzed through the Genotype-Tissue Expression (GTEx) Portal (Lonsdale et al. 2015). (b) mRNA levels of IRX1 was analyzed in the human cancer cell lines (CCLE, cancer cell line encyclopedia, Broad Institute)

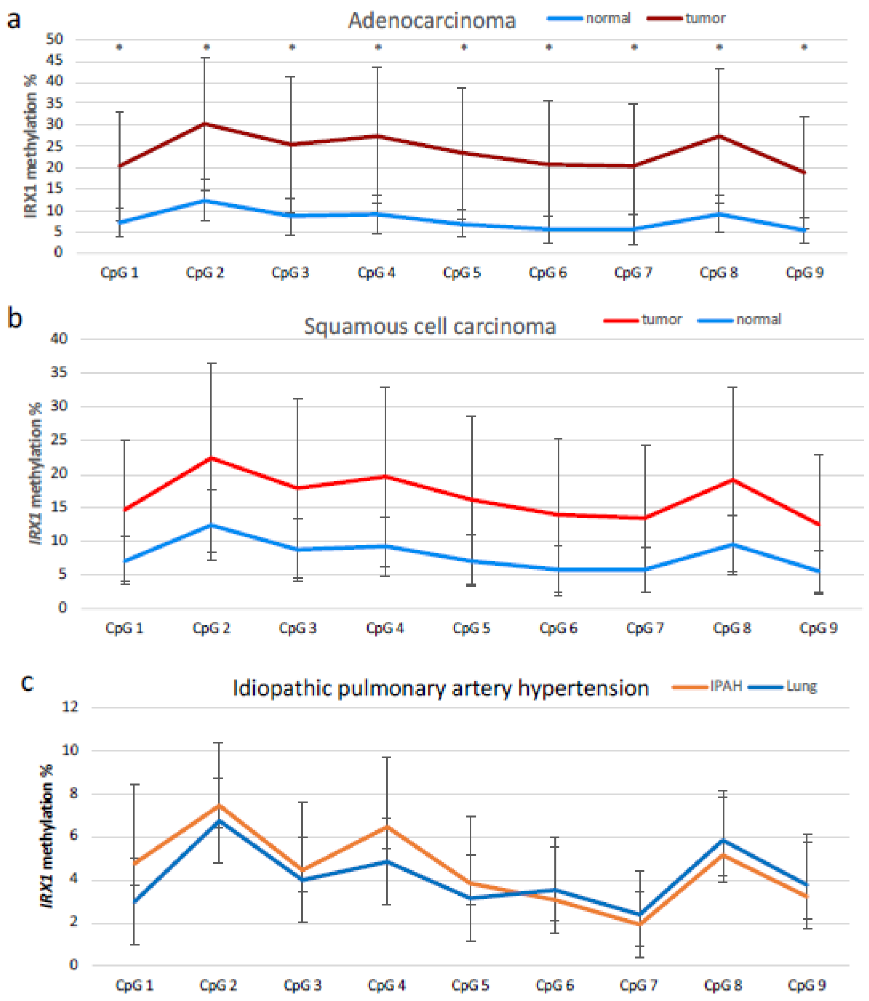

Figure S2: IRX1 promotor methylation of 9 analyzed CpG sites in NSCLC and IPAH patients. (a). Significant tumor specific IRX1 promotor methylation in ADC in 9 analyzed CpG dinucleotids by pyrosequencing of matching pairs of 100 ADC tumor and lung samples. Shown is the mean of all samples analyzed CpG dinucleotids and SD. Stars represent significant increase of tumor methylation compared to normal lung methylation within one CpG. Significance via unpaired t-test p<0.01. (b) Tumor specific IRX1 promotor methylation in SQCC in 9 analyzed CpG dinucleotids by pyrosequencing of matching pairs of 100 SQCC tumor and normal lung samples. (c) IRX1 methylation in 10 IPAH and 8 lung samples by bisulfite pyrosequencing.

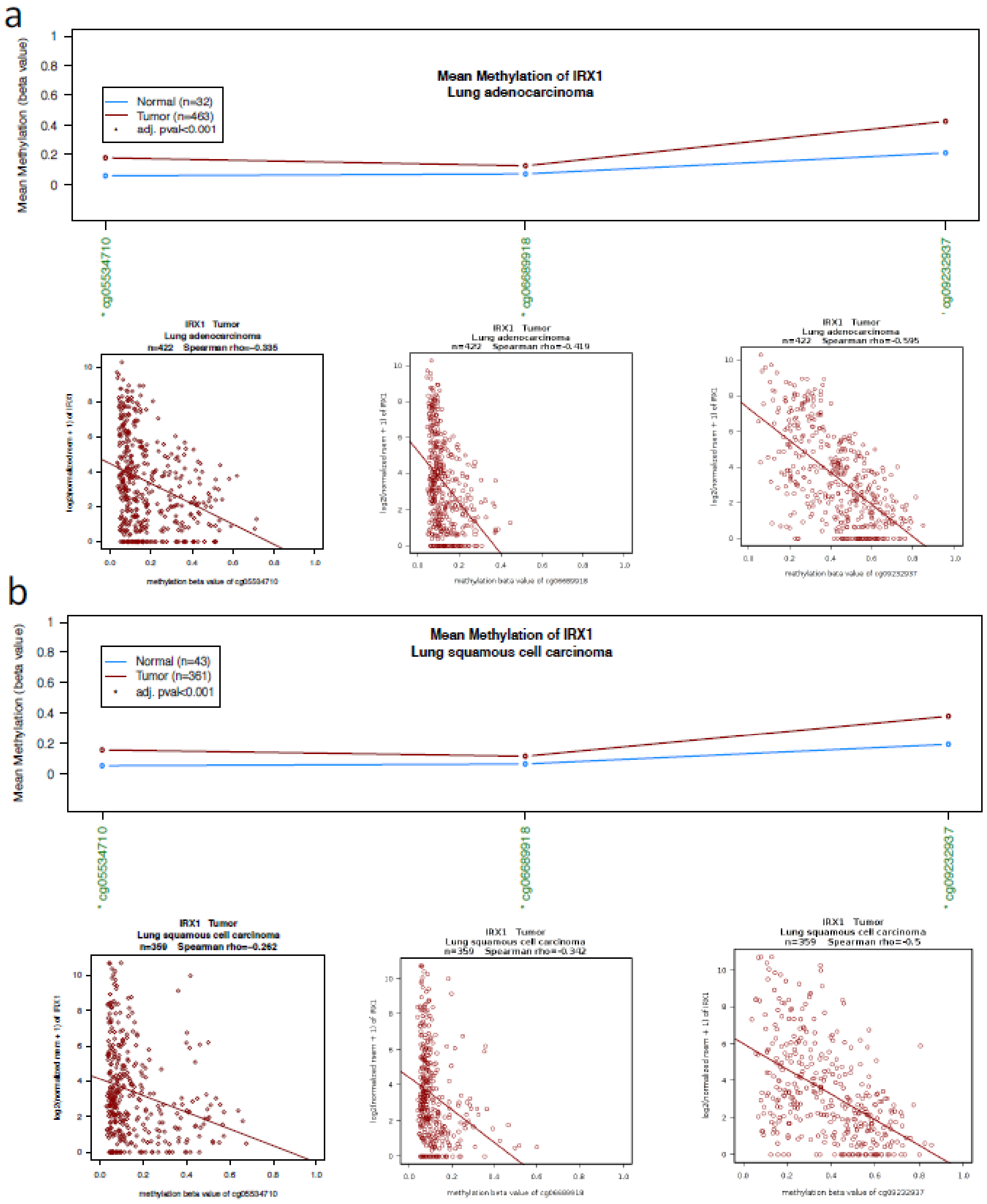

Figure S3. Methylation and expression of IRX1 in ADC (LUAD) and SQCC (LUSQ). (a) Methylation level of the IRX1 promoter at the three CpG sites (cg05534710, cg06689918 and cg09232937) were analyzed for TCGA LUAD samples (463 tumor and 32 normal samples) by TCGA Wanderer Platform (Diez-Villanueva et al., 2015). Hypermethylation was correlated with reduced IRX1 expression. Mean methylation levels are plotted in beta-value (1=100% methylation) and pvalue was calculated by the Wanderer platform. (b) Correlation analysis of IRX1 promoter methylation (beta-value) and IRX1 expression in TCGA LUSQ (361 tumor and 43 normal tissues.

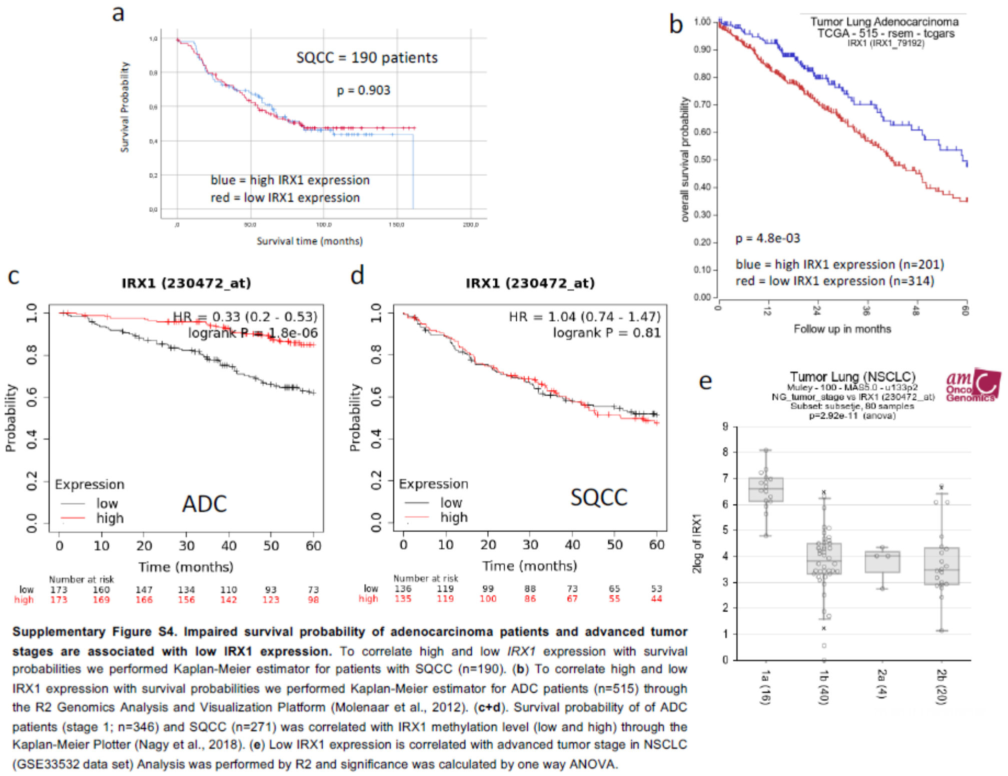

Figure S4. Impaired survival probability of adenocarcinoma patients and advanced tumor stages are associated with low IRX1 expression. To correlate high and low IRX1 expression with survival probabilities we performed Kaplan-Meier estimator for patients with SQCC (n=190). (b) To correlate high and low IRX1 expression with survival probabilities we performed Kaplan-Meier estimator for ADC patients (n=515) through the R2 Genomics Analysis and Visualization Platform (Molenaar et al., 2012). (c+d). Survival probability of of ADC patients (stage 1; n=346) and SQCC (n=271) was correlated with IRX1 methylation level (low and high) through the Kaplan-Meier Plotter (Nagy et al., 2018). (e) Low IRX1 expression is correlated with advanced tumor stage in NSCLC (GSE33532 data set) Analysis was performed by R2 and significance was calculated by one way ANOVA.

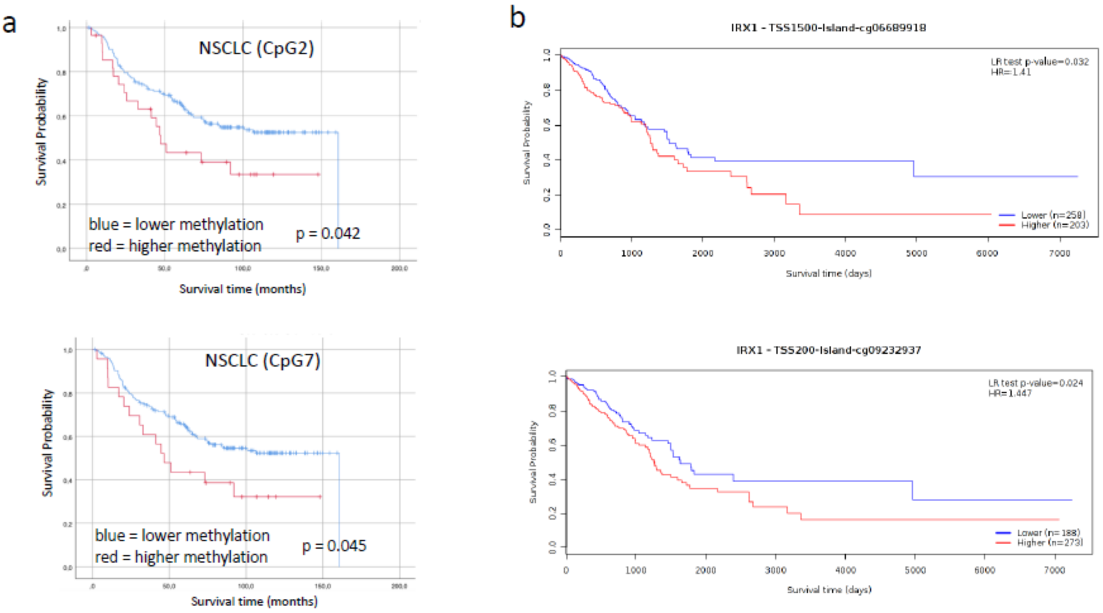

Figure S5. Impaired survival probability of patients with NSCLC is associated higher IRX1 methylation. (a) Survival probability of patients with NSCLC (n=200) is signficantly associated with

methylation of IRX1 at CpG2 and CpG7. (b) Survival probability of patients with ADC (n=461, number of deaths=165) was correlated with IRX1 methylation level at two CpG sites (cg06689918 and cg09232937) by the MethSurv tool (Modhikur et al., 2018). Survival of patients (days) was correlated with lower or higher IRX1 methylation. Significance was calculated by MethSurv (split by maxstat).

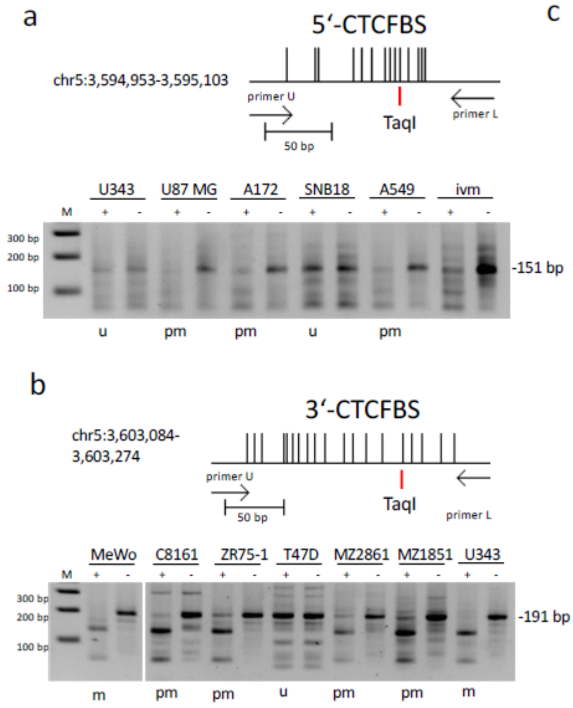

c

tissue	cell line	5'-CTCFBS	3'-CTCFBS	*IRX1*
lung	A549	pm	pm	m
glioblastoma	SNB18	u	pm	u
	U343	u	m	u
	U251	u	m	u
	U118	u	pm	u
	A172	pm	pm	pm
	U87MG	pm	m	pm
breast	ZR-75-1	pm	pm	pm
	T47D	pm	u	u
kidney	HEK293	pm	pm	m
	MZ 2861	pm	pm	pm
	MZ 1851	pm	pm	pm
skin	MeWo	pm	m	pm
	IGR1	pm	pm	pm
	C8161	m	pm	pm

Figure S6. Methylation analysis of the IRX1 5′- and 3′-CTCF binding sites (CTCFBS) in cancer cell lines. Methylation of (a) 5′- CTCFBS and (b) 3′-CTCFBS was analyzed in different human cancer cell lines by CoBRA (151 and 191 bp, respectively). Upper panel shows schematic representation of the analyzed regions on chromosome 5 (hg19), single CpGs (black vertical lines) and TaqI restriction sites (red). Lower panel shows 2% TBE agarose gels with digestion products, together with 100 bp marker (M). Abbreviations are unmethylated (u), partially methylated (pm) and methylated (m), digestion (+), mock digestion (-). An in vitro methylated (ivm) positive control was used. (c) Summary of methylation analysis of the IRX1 locus (promoter, 5′- and 3′-CTCFBS) in different cancer cell lines.

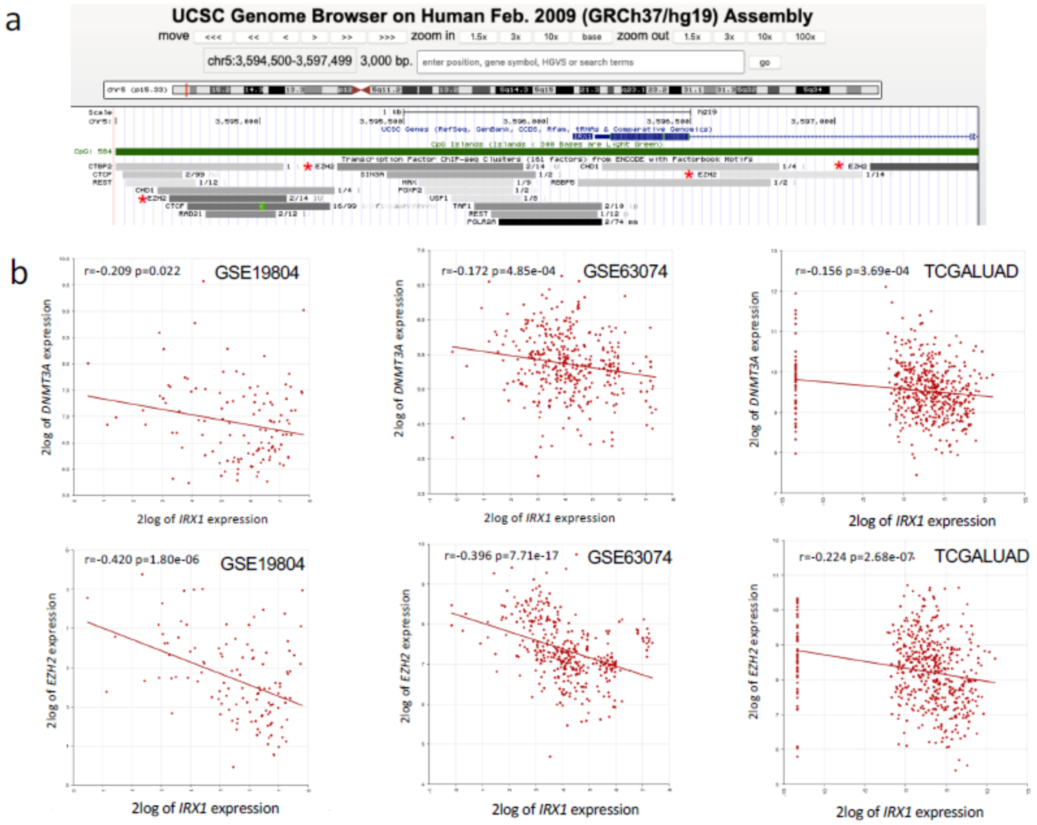

Figure S7. Regulation of IRX1 by EZH2 and DNMT3A. (a) UCSC genome browser data revealed that transcription regulators as EZH2 (red asterisk) bind to a 3 kb region of proximal to the IRX1 promoter as analyzed by ChIP-seq in the ENCODE data set. The CpG island of IRX1 is marked in green and the first exon of IRX1 is shown in blue (b) Significant inverse correlation between IRX1 expression and DNMT3A or EZH2 levels in primary NSCLC. Correlation analysis of IRX1 and DNMT3A or EZH2 expression in primary 120 NSCLC samples (GSE19804 data set), 410 NSCLC (GSE63074 data set) and 515 lung adenocarcinoma (TCGALUAD data set). Analysis was performed by R2 platform (Molenaar et al., 2012).

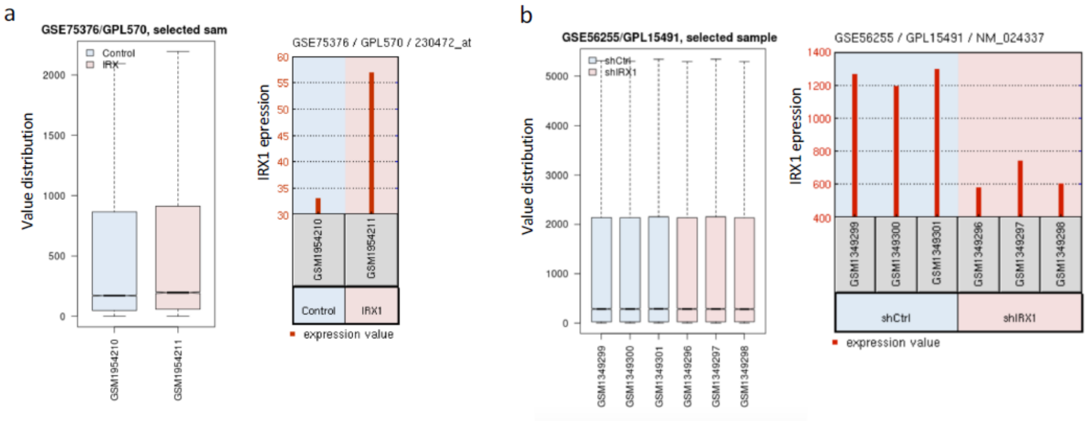

Figure S8. IRX1 expression after IRX1 transfection in HEK293T cells and IRX1 knock down in osteosarcoma 143B cells. (a) IRX expression values (230472_at) were analyzed in pcDNA3.1-GFP (Control) and pcDNA3.1-IRX1-GFP transfected HEK293T cells in the data set GSE75376 (Kühn et al., 2016) by GEO2R (Davis et al., 2007). (b) Expression of IRX1 (NM_024337) was analyzed in 143B cells after IRX1 (shIRX1) and control (shCtrl) knockdown in triplicates in the data set GSE56255 (Lu et al., 2015) by GEO2R.

Supplementary Table S1. Summary of clinicopathological data of NSCLC patients

DNA methylation analysis		N (%)
Age median (range)	66 (40-88) yrs	200 (100%)
Gender	male<br>female	137 (68.5%)<br>63 (31.5%)
Smoking status	current smoker<br>former smoker>6 months<br>former smoker<6 months<br>never smoker<br>n.a.	71 (35.5%)<br>75 (37.5%)<br>35 (17.5%)<br>19 (9.5%)
Tumor stage<br>(UICC TNM ed. 7)	IA<br>IB<br>IIA<br>IIB<br>IIIA	12 (6.5%)<br>61 (30.5%)<br>31 (15.5%)<br>35 (17.5%)<br>60 (30.0%)
Histology	adenocarcinoma<br>squamous cell carcinoma	100 (50%)<br>100 (50%)
ECOG PS	0<br>1<br>2	152 (76.0%)<br>46 (23.0%)<br>2 (1.0%)
Survival status	alive<br>dead	107 (53.5%)<br>93 (46.5%)

Expression analysis		N (%)
Age median (range)	65 (38-88) yrs	416 (100%)
Gender	male<br>Female	288 (69.5%)<br>112 (30.5%)
ECOG PS	0<br>1<br>2	320 (76.9%)<br>89 (21.4%)<br>7 (1.7%)
Smoking status	current smoker<br>former smoker>6 months<br>former smoker<6 months<br>never smoker<br>n.a.	145 (34.9%)<br>149 (35.8%)<br>75 (18.0%)<br>42 (10.1%)<br>5 (1.2%)
Tumor stage<br>(UICC TNM ed. 7)	IA<br>IB<br>IIA<br>IIB<br>IIIA<br>IIIB	46 (11.1%)<br>115 (27.6%)<br>82 (19.7%)<br>56 (13.5%)<br>105 (25.2%)<br>12 (2.9%)
Histology	adenocarcinoma<br>squamous cell carcinoma	226 (54.3%)<br>190 (45.7%)
Survival status	alive<br>dead	207 (49.8%)<br>209 (50.2%)
